# Manifestations of Intellectual Disability, Dystonia, and Parkinson's Disease in an Adult Patient with *ARX* Gene Mutation c.558_560dup p.(Pro187dup)

**DOI:** 10.1155/2023/3636748

**Published:** 2023-02-09

**Authors:** Maria Arvio, Jaana Lähdetie, Hannu Koivu, Antti Sohlberg, Eero Pekkonen

**Affiliations:** ^1^Wellbeing Service County of Päijät-Häme, Lahti, Finland; ^2^PEDEGO, Oulu University Hospital, Oulu, Finland; ^3^Department of Child Neurology and General Practice, Turku University and Turku University Central Hospital, Turku, Finland; ^4^Department of Neurology, Helsinki University Hospital and Department of Clinical Neurosciences (Neurology) University of Helsinki, Helsinki, Finland

## Abstract

We describe a 38-year-old male patient with intellectual disability and progressive motor symptoms who lacked an etiological diagnosis for many years. Finally, clinical exome sequencing showed a likely pathogenic variant of the *ARX* gene suggesting Partington syndrome. His main symptoms were mild intellectual disability, severe kinetic apraxia, resting and action tremor, dysarthria, tonic pupils, constant dystonia of one upper limb, and focal dystonia in different parts of the body, axial rigidity, spasticity, epilepsy, and poor sleep. Another likely pathogenic gene variant was observed in the *PKP2* gene and is in accordance with the observed early cardiomyopathy. Single-photon emission computed tomography imaging of dopamine transporters showed a reduced signal in the basal ganglia consistent with Parkinson's disease. Therapies with a variable number of drugs, including antiparkinsonian medications, have yielded poor responses. Our case report extends the picture of the adult phenotype of Partington syndrome.

## 1. Introduction

Intellectual disability (ID) is a neuropsychiatric manifestation with either a genetic, acquired, or multifactorial background. The clinical picture of an individual with ID is often syndromic. Frequent comorbidities are other neuropsychiatric and neurological impairments such as epilepsy, movement disorders, autism spectrum disorder, and behavioral disturbances as well as somatic disorders such as motor handicaps of varying severity. Of all people with severe ID (IQ < 35), 90% have associated impairments and 10% show a progressive clinical course [1].

Identifying the cause of ID is of great importance for the patient and for his/her family. Firstly, it enables genetic counseling. Secondly, certain health problems manifest themselves in certain ID syndromes, and the exact diagnosis is of great value for the prognosis and treatment of the patient.

Modern genetic techniques have greatly improved and widened the diagnostics of ID etiology but also challenge it because of scarce knowledge related to causal connections between genetic findings and clinical manifestations.

Partington syndrome is a rare X-chromosomal disorder [2–7]. In the literature, the clinical picture is mainly based on descriptions of young patients. Four of the five individuals with ID are adults, and therefore, it is important to publish reports of adult patients with rare syndromes. In the literature, the clinical course of Partington syndrome is assumed to be nonprogressive [2–7].

We describe a 38-year-old male patient who finally, after several consultations and examinations, turned out to have a complex phenotype with multiple, progressive symptoms due to a likely pathogenic variant of the *ARX* gene.

The patient and his mother signed a consent form allowing this publication.

## 2. Case Description

The patient is the second of three children of a nonconsanguineous couple. The 70-year-old mother and two brothers are healthy. From the age of 50, the father suffered from a progressive neurological condition of unknown cause characterized by ataxia, dysarthria, polyneuropathy, and spastic tetraparesis and died at 58 years of age.

The patient was born after an uncomplicated pregnancy at the 39th gestational week. His birth measurements were normal. He spent the first weeks of life in hospital because of apnea and slight muscular hypotonia. During the first years of life, he underwent surgery for bilateral clubfoot and retentive testicles. He learnt to walk at 19 months and said his first words at three years of age. Mild ID was diagnosed before school age with associated dysarthria and tremor mainly on the left side in arms and hands both in action and at rest. Etiological studies, including prometaphase chromosomes and fragile X-DNA revealed no cause of disability. Electroneuromyography (ENMG) showed diffuse polyneuropathy.

At the age of 10, he had a generalized tonic-clonic seizure, and between the ages of 14 to 21 single atonic seizures. An electroencephalogram (EEG) showed generalized slowing and brain magnetic resonance imaging (MRI) an unspecific white matter abnormality near the left ventricle. Sodium valproate-lamotrigine combination therapy had a good response.

Since the age of 27 years, the condition of the patient has shown progression. The first author met him for the first time when the patient was 28 years old. He had temper attacks with nausea, collapse, and redness of the face lasting for a couple of minutes as well as constant resting and action tremors. He wore sunglasses because of tonic mydriatic pupils. Other ophthalmological findings included nystagmus, strabismus, and meibominitis. His gait was clumsy and slow, and he wore orthopedic shoes. As a skin manifestation, there was purpura pigmentosa around both ankles and several fibromas near the toenails. Array-CGH was normal. Brain MRI revealed no signs of white matter progression.

**Figure 1:**
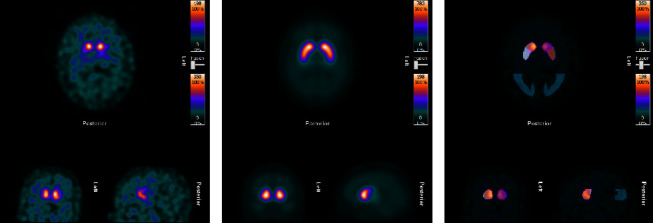
(a) DAT scan images of the patient, (b) mean DAT images of the healthy subjects, (c) striatal region of interest used to calculate SBRs overlaid on the patient scan. Image on the top is a transverse slice, the bottom left is a coronal slice, and the bottom right is a sagittal slice through the striatum. Images illustrate the reduced nigrostriatal dopaminergic activity of the patient.

Between the ages of 30 to 34 years, the patient slept only a few hours daily. The motor symptoms in his hands were now dystonic, with massive clonic jerks when reaching for some objects (limb kinetic apraxia). The patient also suffered from dystonic cramps in his toes and feet, resulting in wounds on the soles. Other dystonic symptoms were head nodding, subcutaneous myokymias in the throat, which were easily observed by outsiders, and facial dystonic movements provoked by shaving. Propranolol and gabapentin gave no response. After the initiation of haloperidol, the dystonic symptoms worsened. At this point, sodium valproate was withdrawn but the dystonic symptoms continued to increase. Escitalopram gave some relief to myokymias and nausea redness attacks. Mirtazapine gave no aid to poor sleep. After the withdrawal of lamotrigine, the patient slept better. A new brain MRI showed no changes. The clinical exome sequencing (CES) identified a variant of the *ARX* gene, a microsatellite insertion of 3 bp in exon 2 causing the addition of a proline in the protein c.558_560dup p. (Pro187dup) [8]. CES of the deceased father (stored tissue) did not reveal pathogenic variants that would have suggested the cause of his disorder [9]. Due to a suspected movement disorder, the patient underwent diagnostic single-photon emission computed tomography (SPECT) imaging of the basal ganglia with a radioactive tracer (DaTscan), Iodine-123-labelled- ioflupane, of the transporter. It showed bilaterally reduced nigrostriatal dopamine transport activity: in the putamen a total lack of the signal and a reduced signal in the nucleus caudatus, consistent with Parkinson's disease [10] (Figure 1). Striatal binding ratios (SBRs) of the patient and site-specific SBR normal values are presented in Table 1.

Pharmacological treatment with oral L-dopa-carbidopa was started at first showing slight improvement in bradykinesia and tremor followed by gradual fading. Maximal daily levodopa dose was 800 mg shared in four doses. Because of the deterioration of the motor symptoms, the levodopa response was assessed by a levodopa challenge test about three years after the initiation of levodopa. A clinically insignificant levodopa response was observed: the UPDRS-III (Unified Parkinson's Disease Rating Scale, [11]) score was 80 points with medication OFF, and 74 points with medication ON about one hour after a single dose of 300 mg of dispersible levodopa. There were no effects on severe bradykinesia, e.g., rising from a chair or gait. Only a slight improvement of resting tremor was observed.

Diagnostic examinations were updated. CES in the Centogene Laboratory revealed two likely pathogenic gene variants. The first was the above-mentioned variant of the *ARX* gene confirming the suspicion of Partington syndrome, the mother being a healthy carrier of this variant. The second abnormality observed was in the *PKP2* gene c.176 A > T, p.(Gln59Leu), which is known to manifest as cardiomyopathy. The gene defect prompted us to perform a cardiac MRI and cardiac echography, which showed an early phase of cardiomyopathy.

At present, the 38-year-old patient manifests Partington syndrome with bradykinesia: a slow and clumsy gait and mild contractures in joints, especially in his ankles. It is difficult for him to initiate movements. Facial dysmorphic features include baldness, hypertelorism, strabismus, and a triangular face. He uses dark glasses. He is friendly and does not show any behavioral disturbances. After the discontinuation of antiepileptic medication, no relapses have occurred. The clinical symptoms of the patient are listed in Table 2.

## 3. Discussion

We conclude that the clinical picture of our patient with multiple and progressive symptoms may be mainly caused by a likely pathogenic variant of the *ARX* gene. His condition fulfills the diagnostic criteria for Partington syndrome. Characteristic dystonic movements of the hands are seen in 63% and dysarthria in 54% of patients. Partington et al. suggested that focal dystonia in association with ID may be diagnostic of this syndrome [7]. This description fits with the present case. He also has early symptoms of cardiomyopathy, most probably due to another mutation, a variant of the *PKP2* gene. In addition to ID, he also presented parkinsonian symptoms such as resting tremor, axial rigidity, and bradykinesia. Parkinsonism-like resting tremor has not been described as a symptom of Partington syndrome. Due to the lack of specific etiology of the multifaceted ID syndrome with comorbid morphological signs and other symptoms, and the unsolved diagnosis of the patient's father, the patient was clinically investigated by several clinics and medical experts. Finally, CES ended the “diagnostic odysseia” showing a variant of *ARX*. Surprisingly, the mother turned out to be a healthy carrier of the same *ARX* gene variant.

*ARX*, the human ortholog of the aristaless gene, is one of the genes mutated in X-linked ID. A pleiotropy of gene variants shows a genotype-phenotype correlation [12]. According to gnomAD, the variant observed in our case, c.558_560dup in exon 2 (reference genome GRCh37/hg19), results in the insertion of one amino acid to the ARX protein (p.Pro187dup) but otherwise preserves the integrity of the reading frame. Its clinical significance is unknown, but two cases of ID have been reported [13]. To be noticed, the variant is in exon 2 in which polyalanine expansions in one of the 2 polyalanine tracts represent the most frequently occurring mutation in the *ARX* gene. In patients with *ARX* mutations, ID ranges from mild to severe.

Lack of structural brain abnormalities in repeated MRI imaging despite many neurological symptoms has been confusing and has not aided diagnostics in the present case. However, a normal brain MRI is typical in Partington syndrome [7]. Basal ganglia involvement in *ARX* patients may manifest as specific grasping [14]. Existing results have found almost complete destruction of cortical GABAergic interneurons in *ARX* syndrome. Previously unreported, our findings indicate that also dopaminergic neurons in the basal ganglia may be damaged in *ARX* syndrome.

Any combination of bradykinesia with resting tremor or rigidity is typical for parkinsonism, and Parkinson's disease is the most common form of Parkinsonism [15]. There are also atypical Parkinsonian disorders like progressive supranuclear palsy, multiple system atrophy, and corticobasal syndrome [16]. These syndromes show, like Parkinson's disease, reduced dopaminergic activity in DATscan. However, the response to levodopa is usually modest, and the disease progresses quite rapidly compared with Parkinson's disease. Given that our patient represented the aforementioned DATscan findings and motor symptoms, Partington syndrome appears to be one rare, atypical Parkinsonian disorder.

The observation of a mutation of the *PKP2* gene known to cause arrhythmogenic right ventricular cardiomyopathy fits with the mild cardiomyopathy of the patient [17]. There is a known founder effect of the observed variant c.176 A > T p.(Gln59Leu) in Finland with 20% penetrance [18]. The mutation is classified as likely pathogenic by ClinVar.

## 4. Conclusion

Next generation sequencing and other methodological achievements have paved the way for understanding the human genome and the etiology of syndromes. Partington syndrome may be manifested with multiple neurological, neuropsychiatric, and somatic signs. An impaired dopamine activity in the striatum together with parkinsonism may be additional signs of Partington syndrome at adult age.

## Figures and Tables

**Table 1:** Striatal binding ratios (SBRs) of the patient scan and site-specific SBR normal values as mean ± standard deviation.

Region of interest (normal values)	SBR patient scan (*Z*-score)
Right caudatus (3.12 ± 0.42)	1.83 (−3.06)
Left caudatus (3.16 ± 0.40)	1.96 (−2.99)
Right putamen (2.95 ± 0.33)	1.07 (−5.68)
Left putamen (2.99 ± 0.40)	1.18 (−4.52)

**Table 2:** Clinical manifestations of the patient with Partington syndrome.

Neurological symptoms	At the age

Clumsiness of gait	Since childhood
Dysarthria	Since childhood
Mild intellectual disability	Since childhood
Tremor in rest and action, mainly of the left arm and hand	Since childhood
Spasticity in lower limbs	Since childhood
Epileptic seizures	Between ages 14–21
Parkinsonism like resting tremor	Since the age of 27
Rigidity	Since the age of 27
Dystonic symptoms such as head nodding, myokymias of the throat, facial movements provoked by shaving	Since the age of 30
Limb kinetic apraxia. Dystonic beating movements when trying to grab objects	Since the age of 30
Cramps in toes resulting in wounds on soles	Since the age of 35

*Psychiatric symptoms*	
Agitation attacks with collapses and nausea and redness of face	Between ages 27–37
Poor sleep	Between ages 30–35

*Eyes*	
Strabismus	Since childhood
Tonic pupils, wears sunglasses	Since childhood

*Skin*	
Bald	Since the age of 25
Purpura pigmentosa	?
Fibromas near toenails	Since the age of 30
Meibomitis	Since the age of 30

*Structural signs*	
Bilateral clubfoot	Since birth, surgical treatment
Retentive testicles	Since birth, surgical treatment
Joint contractures in ankles	Since childhood
Early phase of cardiomyopathy	Diagnosed at the age of 36

## Data Availability

The data supporting the findings of the study are included within the article and are available from the corresponding author upon request.
